# Prevalence of Alzheimer's Dementia and Its Risk Factors in Community-Dwelling Elderly Koreans

**DOI:** 10.4306/pi.2008.5.2.78

**Published:** 2008-06-30

**Authors:** Seok-Ju Choi, Sung-Soo Jung, Young-Sun You, Bae-Seob Shin, Ji-Eun Kim, Sung-Wook Yoon, Dong-Wook Jeon, Jun-Hyung Baek, Sung-Woo Park, Jung-Goo Lee, Young-Hoon Kim

**Affiliations:** 1 Department of Psychiatry, Dong Nam Mental Hospital, Gimhae, Korea.; 2 Department of Psychiatry, Busan Paik Hospital, Inje University College of Medicine, Busan, Korea.; 3 Paik Institute for Clinical Research, Inje University, Busan, Korea.; 4 Department of Psychiatry, School of Medicine and Paik Institute for Clinical Research, Inje University, Busan, Korea.

**Keywords:** Alzheimer's dementia, Mild cognitive impairment, Prevalence, Risk factor, Metabolic syndrome

## Abstract

**Objective:**

We estimated the prevalence of Alzheimer's dementia (AD) and mild cognitive impairment (MCI) and their risk factors in an urban community setting, focusing especially on metabolic syndrome.

**Methods:**

A two-phase investigation based on a door-to-door survey was performed. In Phase I, we administered the Korean version of the Mini-Mental State Examination (MMSE-KC) of the Consortium to Establish a Registry for Alzheimer's disease (CERAD-K). Assessment Packet and the Korean version of the Geriatric Depression Scales (GDS-K) to all 706 participants aged 65 years or older. In Phase II of the study, 175 persons underwent physical and neurological examinations according to the protocol of the CERAD-K clinical assessment battery [CERAD-K (C)] and the neuropsychological assessment battery [CERAD-K (N)]. We also examined the association between cognitive decline and metabolic syndrome. AD and MCI were defined using the DSM-IV-TR criteria and the Clinical Dementia Rating (CDR) scales.

**Results:**

The mean age (±SD) of the subjects was 74.3±16.7 years and the ratio of males to females was 53.2 to 46.8. The prevalence of Alzheimer's dementia was 9.0%, while that of MCI was 32.9%. Old age and lower educational level had significant associations with cognitive decline in the elderly, but gender, years of alcohol intake or smoking, and metabolic syndrome were not associated with AD or MCI.

**Conclusion:**

In this study, metabolic syndrome was not associated with Alzheimer's AD or MCI. Information regarding an association between Alzheimer's dementia and metabolic syndrome in this study will be helpful in formulating future public health policy and prevention strategies in Korea.

## Introduction

Alzheimer's dementia (AD) is a disease which is strongly associated with age, occurring in 1% of those aged 60-65, 6% of those aged 75-79, and 45% of those aged 95 or older.^1^ The prevalence of AD is rising along with the increase in global life expectancy. In Korea, the proportion of people aged 65 years and over is expected to increase from 7.3% in 2000 to 15.1% in 2020.^2^ Because the average age of society is advancing and the number of patients with AD is increasing, it is important to maintain the quality of life in the elderly and to minimize the cost of medications and care by removing as many risk factors as possible for AD and cognitive impairment.^3^ AD is one of the most distressing and burdensome mental health problems in the elderly.^4^

Many studies have been conducted around the world on the prevalence of AD and its risk factors. The prevalence of AD in persons aged 65 and older has been reported to be 3.6-10.3% in Western countries,^5^,^6^ 1.8-4.6% in China, 3.7-6.7% in Japan,^7^ and 9.5-10.8% in Korea.^8^,^9^ The inci dence of AD in cognitively normal individuals over age 65 has generally been reported to be 1-2% per year.^10^

Across the age continuum from middle to later life, the onset of AD is gradual and is believed to be preceded by an earlier state of mild cognitive impairment (MCI). The term, MCI, was originally used to describe an amnestic state, but has since been broadened to include impairments in other cognitive domains.

It is now one of several terms which is used to describe a cognitive state intermediate between normal aging and AD, often with the implication that it is a risk factor or prodromal state for AD or other dementias.^11^

Community-based studies have reported MCI prevalences ranging from 2.8 to 6.1%.^12^,^13^ In the large, nationally representative Canadian Study of Health and Aging, the prevalence of MCI was about 16%, twice that of AD.^14^ In the Cardiovascular Health Study, 6% of the sample had amnestic MCI and 16% had MCI with multiple cognitive deficits^15^; 6% of all MCI cases and 21.5% of all multiple deficit cases did not have memory impairment.13 In the Leipzig Longitudinal Study of the Aged, the prevalence of MCI varied from 3 to 20%, depending on the definition of MCI.^16^ Known risk factors for AD include advanced age, female gender, lower educational level, cerebrovascular disease, hypertension, arrhythmia, hyperlipidemia, alcohol-related disorders, diabetes mellitus, and smoking. All of these can be modified or prevented to some degree.^17^,^18^

While hypertension is known to be a risk factor for cerebrovascular events and vascular dementia, recent studies show that midlife hypertension is also a risk factor for cognitive impairment and AD in later life.^3^,^17^ Cardiovascular and metabolic risk factors, such as hypertension and diabetes, have been hypothesized to play roles in the pathogenesis of AD and in the development of vascular dementia.^19^-^22^

Metabolic syndrome, a clustering of several commonly occurring disorders that includes abdominal obesity, hypertriglyceridemia, low high-density lipoprotein (HDL) level, hypertension, and hyperglycemia, has not been specifically investigated as a risk factor for cognitive impairment in the elderly. Metabolic syndrome may be a risk factor for cognitive impairment simply because it summarizes the combined effects of the risk factors associated with the component disorders.

The prevalence of metabolic syndrome, like that of cognitive disorders, increases with age. If metabolic syndrome is associated with an increased risk of developing cognitive impairment, then early identification and treatment of individuals suffering from metabolic disorder may offer opportunities for modifying the development of cognitive disorders.^23^,^24^

Obesity and sedentary lifestyles have become more common in Korea, just as they have in developed Western countries. It is therefore very important to identify and clarify the deleterious effects of modifiable behaviours in increasing the risk of outcomes, such as cognitive impairment.^24^

This study aimed to estimate the prevalence of AD and mild cognitive impairment and to survey their risk factors, especially metabolic syndrome in older people in Busan, a metropolitan area of Korea.

## Methods

This is a community study, which employed a two-stage design to obtain estimates of the prevalence of AD and its risk factors, especially metabolic syndrome. The study was conducted from July 2005 through February 2007 in Gaegeum 2 dong, Busanjin-gu, one of the 16 districts in Busan, the second largest city in Korea. The total population of Gaegeum 2 dong was 16,817, and 1,215 (7.22%) residents were aged 65 and older (established from resident registration records in January 2005).

The institutional review board of the Medical College of Inje University, Busan Paik Hospital, Korea, approved the study protocol. All participants or family members provided informed consent.

### Phase I: Population survey

During the period July 2005 through February 2006, 1,215 elderly people were invited to participate in a survey, which involved them responding to a structured interview administered in their homes by two trained research nurses and seven psychiatrists. If the respondent could not provide sufficient information without assistance, reliable informants (spouse, child, other relatives, close friends, in that descending order) were also interviewed. The structured interview consisted of demographic questions, past and present medical history, the Mini-Mental State Examination (MMSE-KC) of the Korean version of the Consortium to Establish a Registry for Alzheimer's Disease Assessment Packet (CERAD-K),^25^ and the Korean version of the Geriatric Depression Scale (GDS-K).^26^

The demographic part of the structured interview consisted of questions on age, gender, education, height, weight, family members living with the subject, and previous or present occupation. The medical history involved questions relating to hypertension, diabetes mellitus, stroke, major endocrine/hepatic/pulmonary/renal/cardiac disease, head trauma, seizures, encephalitis, Parkinson's disease, alcohol abuse, depression, other major psychiatric illnesses, and the use of prescription and/or nonprescription medications.

### Phase II: Clinical evaluation

Each of the Phase I participants was placed in one of three groups according to performance on the MMSE-KC: 'good' (MMSE-KC, 0-1.0 SDs below the mean score), 'intermediate' (MMSE-KC, 1.0-1.5 SDs below the mean score), or 'poor' (MMSE-KC≧1.5 SDs below the mean score).

For the Phase II clinical evaluation, participants were then randomly sampled from each group using different sampling fractions. The group-specific sampling fractions were 20% for the 'good', 50% for the 'intermediate', and 100% for the 'poor' performance groups. These sampling fractions were designed to increase the number of AD cases included in the study by giving greater weight to the selection of participants from the lower performing groups.

The research physicians involved were blinded to the results of the survey in Phase I.

The clinical evaluations were carried out from September 2006 to February 2007. by a psychiatrist with advanced training in AD and eight psychiatric residents, who conducted standardized clinical interviews and performed physical and neurological examinations according to the protocol of the Korean version of the CERAD Clinical Assessment Battery [CERAD-K (C)].^25^

Where necessary, reliable informants were interviewed to obtain accurate information about cognitive and functional changes and the medical histories of the participants. We depended more heavily on the reports of informants than the test scores in evaluating illiterate or poorly educated subjects. We also used the medical and psychiatric history data and GDS-K^26^ scores collected in the Phase I survey as reference information for the evaluation of each Phase II participant. This made it possible to save time and reduce the possible omission of major medical or psychiatric histories that might have affected the cognitive state of the participants.

Two trained research nurses administered the eight neuropsychological tests from the Korean version of the CERAD neuropsychological assessment battery [CERAD-K (N)].^25^ These were the Verbal Fluency Test, the 15-item Korean Version of the Boston Naming Test, the MMSE-KC, and the tests of Word List Memory, Constructional Praxis, Word List Recall, Word List Recognition, and Constructional Recall.

The CERAD-K (C) and CERAD-K (N) were developed by translating the original English-language version of the CERAD assessment packet^27^ into Korean after considering cultural and linguistic differences. The cognitive tests in CERAD-K (C) and CERAD-K (N) have been shown to be reliable and valid equivalents of the tests in the English-language version of the CERAD clinical and neuropsychological assessment batteries.^25^

All participants in the Phase II evaluation were assessed for metabolic syndrome. The presence of metabolic syndrome was determined with using the National Cholesterol Education Program Third Adult Treatment Panel guidelines, which require the presence of at least three of the following: waist measurement of over 88 cm for women and over 102 cm for men, hypertriglyceridemia [≥150 mg/dL (≥1.69 mmol/L)], low HDL cholesterol [<40 mg/dL (<1.03 mmol/L) in men and <50 mg/ dL (<1.29 mmol/L) in women], high blood pressure (systolic, ≥130 mmHg; diastolic, ≥85 mmHg, using the average of two seated measurements or current use of an antihypertensive medication), and high fasting glucose [≥110 mg/dL (≥6.10 mmol/L) or current use of an antidiabetic (insulin or oral agents) medication]. Lipid levels were measured after fasting.

### Diagnostic criteria and assessments

The presence of AD was initially identified according to the diagnostic criteria in the Diagnostic and Statistical Manual of Mental Disorders, Fourth edition-Text Revision (DSM-IV-TR).^28^ Those Phase II respondents meeting the DSM-IV-TR diagnostic criteria for AD were then further assessed to determine diagnostic subtypes. AD was diagnosed according to the criteria of the National Institute of Neurological and Communicative Disorders and Stroke and the Alzheimer's Disease and Related Disorders Association (NINCDS-ADRDA).^29^ The diagnosis of vascular dementia (VD) was made according to the criteria of the National Institute of Neurological and Communicative Disorders and Stroke-Association Internationale pour la Recherche et l'Enseignement en Neurosciences (NINDS-AIREN) criteria.^30^ A Modified Hachinski Ischemic Score^31^ was also obtained for each case of dementia for reference purposes. All subjects with VD detected in this study scored greater than 4 on the Modified Hachinski Ischemic Scale. In this study, subjects with a probable or possible AD based on each set of diagnostic criteria were included, but those with VD were excluded.

The diagnosis of MCI was established by evidence of memory impairment, preservation of general cognitive and functional abilities, and the absence of diagnosed AD. MCI is staged clinically at the 0.5 severity level on the Clinical Dementia Rating (CDR) scale.^32^ The CDR scale is a dementia staging instrument used to rate the severity of cognitive impairment at five levels from none to a maximal 3 (rated as 0, 0.5, 1, 2, or 3) in each of six domains: memory, orientation, judgment and problem solving, function in community affairs, home and hobbies, and personal care with the last having no 0.5 impairment level.^32^,^33^

Based on collateral sources and an interview of the study subject, a global CDR score was derived from the individual ratings in each domain, so that a CDR-0 indicated no dementia and CDRs 0.5, 1, 2, and 3 represented very mild, mild, moderate and severe dementia, respectively. The CDR-0.5 level was originally designated "questionable dementia or MCI".^33^,^35^

A panel composed of eight psychiatrists reviewed all the data, including the raw materials from the clinical interviews, thephysical and neurological examinations, the neuropsychological test results, and the medical records. They then assigned the final diagnosis and CDR index^35^ and divided all participants into three groups: healthy controls, and patients with MCI or AD.

### Statistical analyses

ANOVA or t-tests were conducted to analyse group differences in terms of the demographic and metabolic syndrome data.^17^,^18^ Univariate adjusted logistic regression analyses were used to explore the separate associations of age, education, weight and GDS-K score with the presence of AD. The possible overestimation of the odds ratios was corrected by adjusting to approximate risk ratios, using the method of Zhang and Yu,^36^ The 95% confidence intervals (CIs) were derived using the exact methods of a binomial parameter. A lack of overlap of the 95% CIs was used as the criterion for a statistically significant difference between any two prevalences. All analyses were conducted using SAS software (version 8.2; SAS Institute, Cary, NC).

## Results

Of the 1215 persons originally contacted, 706 (58.1%) participated in the Phase I population survey. And of these 252, 281, and 130 were assigned to the the good, intermediate and poor performance groups, respectively, according to their MMSE-KC score. Forty-three were excluded from the assignment due to incomplete information. The MMSE-KC mean score (±SD) of all the Phase I participants was 22.1±7.2. The medical and psychiatric history data and GDS-K scores collected in the Phase I interview were used only as reference information in the Phase II clinical evaluation of each participant, and quantitative summaries of these data are not provided in this report. The reasons for the nonparticipation in the Phase I survey by the remaining 509 people were as follows: refusal (102 persons, 20.0%), incorrect address or change of address (327 persons, 64.2%), not being home for three attempted visits (40 persons, 7.9%), serious illness (30 persons, 5.9%), and death (10 persons, 2.0%). Of the 706 participants, 267 from the three MMSE-KC score groups were invited to participate in the Phase II clinical evaluation, and 175 (65.5%) completed the evaluation. The reasons for noncompletion of the Phase II clinical evaluation by the remaining 92 were as follows: refusal (43 persons, 47.1%), not being home for three attempted visits (29 persons, 31.5%), admission for serious illness (16 persons, 17.4%), and death (4 persons, 4.35%).

**FIGURE 1:**
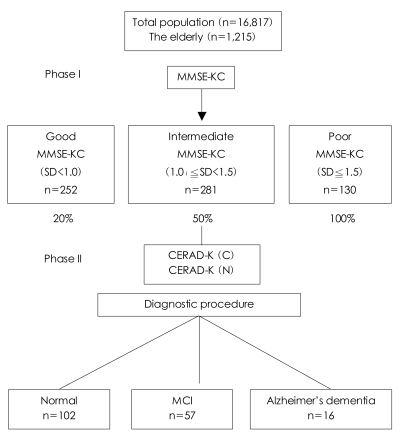
Summary of the study participants. MMSE-KC: Mini-Mental State Examination-KC, CERAD-K: Consortium to Establish a Registry for Alzheimer's disease-K, MCI: mild cognitive impairment.

The subjects who did not participate in the Phase I survey or the Phase II clinical evaluation were not significantly different in terms of age, education, or gender from those who did participate. The mean interval (±SD) between the completion of Stage I and Phase II was 394±48 days. The mean age (±SD) of the subjects was 74.3±16.7 years, and the ratio of males to females was 53.2 to 46.8. Of the 175 (65.5%) people who participated in the Phase II clinical evaluation, 16 met the DSM-IV-TR criteria for AD, 57 were rated as having MCI^23^ and 102 had normal cognitive functioning (Figure 1). The overall prevalences, adjusted for those aged 65 and older in the population of Gaegeum 2 Dong, Busanjin-gu, were estimated to be 9.0% for AD, 32.9% for MCI, and 58.1% for normal cognitive functioning.

The sociodemographic and clinical characteristics of the subjects participating in the Phase II clinical evaluation are summarized in Table 1. The prevalence of MCI and AD and showed a statistically significant increase with advancing age, lower levels of education, lower MMKE-KC scores, and higher GDS-K scores.

The mean blood glucose and lipid levels and the prevalences of metabolic syndrome in the normal participants and subjects with MCI and AD are shown in Table 2. The overall prevalence of metabolic syndrome in this study was 24.7% (25% in the normal cognitive group, 23.3% in the MCI group, and 30% in the AD group). We found no statistically significant differences in the mean scores for each metabolic syndrome factor and the prevalences of metabolic syndrome in the normal, MCI, and AD groups.

To explore the separate associations of age, years of education, weight, waist circumference, and GDS-K score with the frequency of AD, odds ratios were calculated by a univariate logistic regression analysis. Older age, lower education, higher weight, and higher GDS-K scores were significantly associated with an increased prevalence of AD (Table 3).

## Discussion

The prevalences of AD and MCI were estimated in elderly people in a metropolitan area of Korea, and the independent association of risk factors for AD, especially metabolic syndrome, was explored. The overall prevalence, adjusted for the older population of Gaegeum 2 dong, Busanjin-gu, was estimated to be 9.0% for AD and 32.9% for MCI. The results of this study suggest that old age and low educational level are risk factors for AD and MCI, and that in this study population, metabolic syndrome had no significant association with AD or MCI.

The estimated prevalence of AD of 9.0% is similar to that from a study in Seoul.^7^ However, lower prevalences have been found in urban populations in Japan and China.^37^,^40^,^41^

The estimated prevalence of MCI was 32.9%. The reported prevalences and incidences of MCI or predementia syndromes vary from study to study because of the different diagnostic criteria used and different sampling and assessment procedures. MCI is thought to be a prodromal phase of AD and therefore highly predictive of subsequent "conversion".^42^ In an Italian study, the prevalence was 9.5% for cognitive impairment [not demented (CIND)] and 16.1% for MCI.^43^

Consistent with other studies,^40^,^41^,^44^ the prevalence of AD and MCI increased rapidly with advancing age. Many studies have reported old age, low educational level, female gender, a family history of AD, and a history of head trauma to be risk factors for AD.^44^ Our findings also pointed to old age and low educational level being significant risk factors in the development of AD and MCI (Table 1).

Epidemiologic data supporting the cognitive reserve hypothesis include observations that higher educational and occupational attainment is associated with a decreased risk of AD. Factors other than education and occupation might also provide a reserve against the pathologic changes occurring in AD. More highly educated persons may have a greater cognitive reserve, and this may postpone the clinical manifestations of AD.^45^-^48^

Many studies have also reported that AD was more prevalent in women, while a few have reported that the prevalence was higher in men, or at least equal between the two genders.^48^,^49^ Another Korean study showed that the overall frequency of AD in women was twice that in men, and the gender difference in the prevalence of AD was statistically significant.^9^ In the present study, however, we found no gender difference.

We explored the separate associations of each factor of metabolic syndrome with the prevalence of AD and MCI. However, neither metabolic syndrome nor any of its component factors were associated with an increased frequency of AD or MCI (Table 2). These results were similar to those of two other prospective studies that found no association between lipoprotein levels and the risk of AD.

Metabolic syndrome also appears to predispose people to cognitive dysfunction and AD.^50^ In elderly women, metabolic syndrome may be an important contributor to the worsening of memory, an essential feature of MCI.^51^ The findings of studies that have investigated the relationship between serum lipoprotein levels and the risk of cognitive impairment are conflicting. Some cross-sectional studies suggest that high total cholesterol levels are associated with an increased risk of AD, while others report that low total cholesterol levels are associated with the rvisk of developing AD.^52^-^54^ In one study of more than 1,000 elderly people, a higher baseline lowdensity lipoprotein (LDL) cholesterol level was associated with an increased risk of developing stroke-related dementia, but not with the risk of developing AD.^55^,^56^

Another study reported a statistically significant association between lower total cholesterol levels and higher rates of cognitive decline in older populations, but its clinical significance remains unclear.^57^,^58^ In an Italian study of elderly people, those who converted from MCI to AD tended to have higher serum HDL levels and lower serum folate levels.^42^

Even when using the same diagnostic criteria no clear agreement exists among clinicians and researchers regarding the degree of functional impairment that is sufficient to warrant a diagnosis of AD.^7^ This issue may be one of the major reasons for the varying estimates of AD across different populations. Our results were based on systematic clinical evaluations and the Korean version of the CERAD battery, including some psychometric tests, thus potentially improving the discrimination between AD, MCI, major depression, and healthy controls.^7^,^19^

To reduce false-negatives, eight trained and experienced psychiatrists and two trained research nurses performed face-to-face clinical diagnostic evaluations of all participants assessed in Phase I and Phase II.

Although care was taken to avoid misdiagnosing AD because of a low level of education or intellectual disability, overall response rates in this study were 58.1% of the total population in the Phase I survey and 24.8% in the Phase II clinical evaluation. The response rate for Phase I was somewhat lower than that of a rural study in Korea (85.2% in Suh et al.^53^). However, it was somewhat higher than the response rates found in other urban studies (37.7% in Cho et al.^36^ and 53.8% in Bae et al.^37^).

The possibility exists that the prevalences of AD and MCI were underestimated in this study. A major cause of the low response rate was the long interval between the Phase I and Phase II evaluations. As in previous studies, people who lived in an affluent residential area frequently refused to participate in the study despite three visits for enrollment.^38^,^39^

Clearly, this study has some limitations. First, the study included only noninstitutionalized elderly people. If institutionalized people had been included, it is likely that the estimated prevalence of AD would have been higher. However, this may be of little importance because institutional care for AD patients is not common in Koreawhere most AD patients live with their families. Secondly, AD was confirmed according to DSM-IV-TR and NINDS-ADRDA criteria. If subjects with a diagnosis of VD were excluded by the panel and did not contribute the estimated prevalence rate of AD. Furthermore, neuroimaging data were not available for all the AD patients. All of these factors may have contributed to an underestimation of the frequency of AD.

The prevalences for AD and MCI were estimated to be 9.0% and 32.9% respectively when adjusted for the older population of Gaegeum 2 dong, Busanjin-gu. The results suggest that whereas old age and low educational level are risk factors for AD and MCI, metabolic syndrome had no significant association with either AD or MCI. Further longitudinal, prospective community studies are needed to test the hypothesis that the epidemiological transition into AD is causally related to modifiable risk factors in the elderly.

## Figures and Tables

**TABLE 1:**
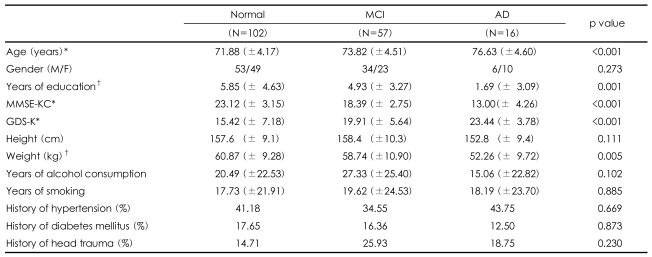
Sociodemographic and clinical characteristics of the subjects

^*^p<0.001, ^†^p<0.01. MMSE-KC: Korean version of the Mini-Mental Status Examination in the Consortium to Establish a Registry for Alzheimer's disease (CERAD) assessment packet. MCI: mild cognitive impairment, AD: Alzheimer's dementia, GDS-K: Geriatric Depression Scales

**TABLE 2:**
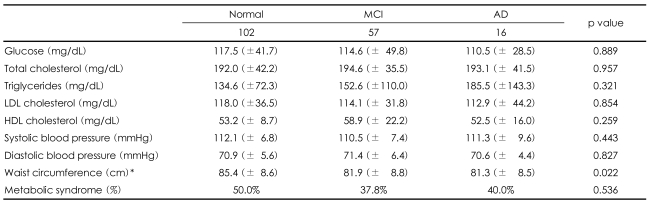
Mean blood glucose and lipid levels and prevalence of metabolic syndrome in normal participants and subjects with MCI and AD

MCI: mild cognitive impairment, AD: Alzheimer's dementia, LDL: low-density lipoprotein, HDL: high-density lipo-protein

**TABLE 3:**
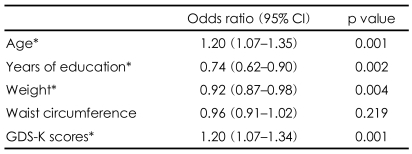
Correlation of significant risk factors with AD

AD: Alzheimer's dementia, GDS-K: Korean version of the Geriatric Depression Scale
